# Roles of Three HSF Domain-Containing Proteins in Mediating Heat-Shock Protein Genes and Sustaining Asexual Cycle, Stress Tolerance, and Virulence in *Beauveria bassiana*

**DOI:** 10.3389/fmicb.2018.01677

**Published:** 2018-07-25

**Authors:** Gang Zhou, Sheng-Hua Ying, Yue Hu, Xiang Fang, Ming-Guang Feng, Jie Wang

**Affiliations:** ^1^College of Food Science, South China Agricultural University, Guangzhou, China; ^2^Guangdong Open Laboratory of Applied Microbiology, Guangdong Provincial Key Laboratory of Microbial Culture Collection and Application, State Key Laboratory of Applied Microbiology Southern China, Guangdong Institute of Microbiology, Guangzhou, China; ^3^Institute of Microbiology, College of Life Sciences, Zhejiang University, Hangzhou, China

**Keywords:** entomopathogenic fungi, heat shock transcription factor, heat shock proteins, gene expression and regulation, asexual development, multiple stress responses, virulence

## Abstract

Heat-shock transcription factors (HSFs) with a HSF domain are regulators of fungal heat-shock protein (HSP) genes and many others vectoring heat-shock elements, to which the domain binds in response to heat shock and other stress cues. The fungal insect pathogen *Beauveria bassiana* harbors three HSF domain-containing orthologous to Hsf1, Sfl1, and Skn7 in many fungi. Here, we show that the three proteins are interrelated at transcription level, play overlapping or opposite roles in activating different families of 28 HSP genes and mediate differential expression of some genes required for asexual developmental and intracellular Na^+^ homeostasis. Expression levels of *skn7* and *sfl1* largely increased in Δ*hsf1*, which is evidently lethal in some other fungi. Hsf1 was distinct from Sfl1 and Skn7 in activating most HSP genes under normal and heat-shocked conditions. Sfl1 and Skn7 played overlapping roles in activating more than half of the HSP genes under heat shock. Each protein also activated a few HSP genes not targeted by two others under certain conditions. Deletion of *sfl1* resulted in most severe growth defects on rich medium and several minimal media at optimal 25°C while such growth defects were less severe in Δ*hsf1* and minor in Δ*skn7*. Conidiation level was lowered by 76% in Δ*skn7*, 62% in Δ*sfl1*, and 39% in Δ*hsf1*. These deletion mutants also showed differential changes in cell wall integrity, antioxidant activity, virulence and cellular tolerance to osmotic salt, heat shock, and UV-B irradiation. These results provide a global insight into vital roles of Hsf1, Sfl1, and Skn7 in *B. bassiana* adaptation to environment and host.

## Introduction

Fungal heat-shock proteins (HSPs) classified to different families by molecular sizes enable to protect cells from stress damages (Hjorth-Sørensen et al., 2001). Their coding genes bear a typical heat-shock element (HSE), i.e., a tandem array of three oppositely oriented DNA consensus motifs (nGAAn), and can be activated by heat-shock transcription factors (HSFs) that bind specifically to the HSE in response to external stress cues (Lindquist, 1986; Lindquist and Craig, 1988; Morimoto et al., 1992; Thompson et al., 2008). In *Saccharomyces cerevisiae*, Hsf1 is an HSF required for not only expression of basal HSP genes, such as *SSA1* and *SSA4* in the Hsp70 family, in response to stress cues but also a variety of cellular events (Sorger and Pelham, 1988; Smith and Yaffe, 1991; Gallo et al., 1993; Andrew et al., 1995). In *Candida albicans*, Hsf1 was shown to regulate expression of *hsp104*, *hsp90*, and *hsp70* genes under normal and stressful conditions, and its mutants lacking the negative regulatory domain CE2 (residues 535–550) became thermosensitive and less virulent (Nicholls et al., 2009, 2011). Inactivation of Hsf1 in *Coniothyrium minitans* resulted in reduced conidiation and decreased tolerance to thermal and osmotic stresses (Hamid et al., 2013).

Aside from Hsf1, Sfl1 also bears a DNA-binding domain (i.e., HSF domain) in yeast (Fujita et al., 1989) and has been identified as a key activator of *hsp30* genes under basal and stressful conditions (Galeote et al., 2007). The HSF domain is crucial for Sfl1 to bind specifically to HSE with an inverted DNA repeat region (AGAA-n-TTCT) (Conlan and Tzamarias, 2001). Deletion of *sfl1* in *C. albicans* resulted in flocculated clumps in cells, enhanced filamentous growth in several media, and depressed expression of *hsp30* and *hsp90* genes under a heat shock (Bauer and Wendland, 2007; Li et al., 2007; Zhang et al., 2008). Deletion of *sfl1* in *Magnaporthe oryzae* led to suppressed expression of *hsp30* and *hsp98* genes, invasive growth, elevated thermosensitivity, and attenuated virulence (Li et al., 2011). Hsf1 and Sfl1 (Hsf2) were proven to be essential for hyphal growth and asexual development in *Neurospora crassa* (Thompson et al., 2008).

Skn7 is another stress-responsive transcription factor that contains the HSF domain and is highly conserved in fungi (Brown et al., 1993). In yeast, Skn7 and Hsf1 may co-activate *hsp12*, *hsp26*, *hsp70*, *hsp82*, and *hsp104* genes in response to oxidative stress so that deletion of *skn7* caused increased sensitivity to oxidative and heat stresses (Morgan et al., 1997; Lee et al., 1999; Raitt et al., 2000). In other fungi, Skn7 is vital for cellular responses to oxidative, osmotic, and cell wall perturbing stresses (Fassler and West, 2011; Hussain et al., 2016) but not necessarily important for pathogenicity. For instance, Skn7 was evidently required for the virulence of *Alternaria alternata* (Chen et al., 2012) and *Metarhizium robertsii* (Shang et al., 2015) but not for the virulence of *M. oryzae* (Motoyama et al., 2008), *Batrytis cinerea* (Yang et al., 2015), and *Fusarium graminearum* (Jiang et al., 2015).

Above all, Hsf1 and Sfl1 mainly regulated the fungal growth, conidiation, thermotolence, and virulence, while Skn7 played important roles in the adaptation to various stress conditions, including oxidative, osmotic stresses, cell wall damage agents, and/or virulence and all of them may take part in controlling the expression of several heat shock proteins in previous studied (**Table 1**). Meanwhile, fungal growth, development, stress tolerance, and virulence regulated by Hsf1, Sfl1, and Skn7 are phenotypes crucial for the pest control potential of filamentous fungal insect pathogens, such as *Beauveria bassiana* widely applied in biological control programs of arthropod pests (Wang and Feng, 2014; Ortiz-Urquiza et al., 2015). The genomic database of *B. bassiana* (Xiao et al., 2012) has Hsf1, Sfl1, and Skn7 orthologs and 28 HSPs, which fall into the small HSP, Hsp40, Hsp60, Hsp70, Hsp90, and Hsp100 families with each containing 1–15 members. However, either three HSF or most HSP genes remain functionally unexplored because only two *hsp40* genes, i.e., *mas5* and *mdj1*, have been characterized in *B. bassiana* (Wang et al., 2016, 2017). It is unclear whether Hsf1, Sfl1, and Skn7 activate different families of HSP genes in an independent or cooperative manner. This study seeks to explore transcriptional linkages of Hsf1, Sfl1, and Skn7 with all 28 HSP genes in *B. bassiana* and to elucidate their functions by multi-phenotypic analyses of deletion/complement mutants. Our results provide a global insight into differential roles for Hsf1, Sfl1, and Skn7 in activating different families of HSP genes and sustaining asexual cycle, virulence, and multiple stress tolerance in *B. bassiana*.

**Table 1:** Changes in various phenotypes caused by disrupted *hsf1*, *sfl1*, and/or *skn7* in fungi.

Mutant	Phenotype	Reference
Δ*hsf1*	Lethality in *S. cerevisiae* and *N. crassa*	Wiederrecht et al., 1988; Thompson et al., 2008
	Reduction in conidiation and decreased tolerance to osmotic stresses and oxidative stresses in *C. minitans*	Hamid et al., 2013
	Thermosensitive and less virulent in *C. albicans* and *C. minitans*	Nicholls et al., 2011; Hamid et al., 2013
	Depressed expression of *hsp90*, *hsp70*, and *hsp104* in *C. albicans*	Nicholls et al., 2011
	**Defective in hyphal growth, conidiation, conidial germination under high temperature, and cell wall integrity; increased sensitivities to oxidative stress and NaCl; attenuated virulence and affected the transcript changes of *hsp20*, *hsp30*, *hsp40*, *hsp70*, and *hsp104* under normal condition and heat shock in *B. bassiana***	**In this study**
Δ*sfl1*	The formation of flocculated clumps in cells in *C. albicans*	Bauer and Wendland, 2007; Zhang et al., 2008
	Enhanced filamentous growth in *C. albicans* but reduced the production of aerial hyphae in *M. oryzae*	Bauer and Wendland, 2007; Li et al., 2007, 2011; Zhang et al., 2008
	Reduction in conidiation and elevated thermosensitivity in *M. oryzae* and *N. crassa*	Thompson et al., 2008; Li et al., 2011
	Attenuated virulence in *C. albicans* and *M. oryzae*	Li et al., 2007, 2011
	Depressed expression of *hsp30*, *hsp90*, and *hsp98* in *S. cerevisiae*, *C. albicans*, *N. crassa*, and *M. oryzae* and increased expression of hypha-specific genes, such as *ECE1*, *HWP1*, *ALS1*, *ALS3*, and *Flo8* in *C. albicans*	Bauer and Wendland, 2007; Galeote et al., 2007; Li et al., 2007, 2011; Thompson et al., 2008; Zhang et al., 2008
	**Defective in hyphal growth, conidiation, conidial germination under normal condition, and cell wall integrity; increased sensitivities to oxidative stresses and NaCl; attenuated virulence and affected the transcript changes of *hsp20*, *hsp40*, *hsp70*, and *hsp104* under normal condition and heat shock in *B. bassiana***	**In this study**
Δ*skn7*	Minor inhibition of hyphal growth in *F. Graminearum*, but not in *H. minnesotensis*, *B. cinerea*, and *A. flavus*	Jiang et al., 2015; Viefhues et al., 2015; Hussain et al., 2016; Zhang et al., 2016
	Defective in conidiation in *F. graminearum*, *H. minnesotensis*, *A. flavus*, and *B. cinerea*	Jiang et al., 2015; Yang et al., 2015; Hussain et al., 2016; Zhang et al., 2016
	Increased sensitivity to high temperature in *S. cerevisiae*, *H. minnesotensis*, but not in *M. robertsii*	Raitt et al., 2000; Shang et al., 2015; Hussain et al., 2016
	Decreased tolerance to oxidative stresses in *S. cerevisiae*, *B. cinerea*, *H. minnesotensis*, *A. flavus*, but increased the tolerance in *M. robertsii*	Lee et al., 1999; Raitt et al., 2000; Shang et al., 2015; Viefhues et al., 2015; Yang et al., 2015; Hussain et al., 2016; Zhang et al., 2016
	Elevated sensitivity to osmotic stresses in *B. cinerea* but not in *M. robertsii*	Shang et al., 2015; Viefhues et al., 2015
	Destroyed cell wall integrity in *B. cinerea*, *A. flavus*, *C. neoformans*, *A. fumigatus*, and *M. robertsii*	Fassler and West, 2011; Shang et al., 2015; Viefhues et al., 2015; Zhang et al., 2016
		
	Attenuated virulence in *C. albican*, *A. alternata* and *M. robertsii*, but not impaired fungal virulence in *M. oryzae*, *B. cinerea*, *F. graminearum*	Singh et al., 2004; Motoyama et al., 2008; Chen et al., 2012; Jiang et al., 2015; Yang et al., 2015; Shang et al., 2015
	Depressed expression of *hsp12*, *hsp26*, *hsp70*, *hsp82*, and *hsp104* in yeast responding to oxidative stresses	Lee et al., 1999; Raitt et al., 2000
	**Minor inhibition of hyphal growth; defective in conidiation, conidial germination under normal condition and UV-B irradiation, and cell wall integrity; increased sensitivities to oxidative stresses, but decreased the sensitivity to NaCl; attenuated virulence and affected the transcript changes of *hsp30*, *hsp40*, *hsp70*, *hsp90*, and *hsp78* under normal condition and heat shock in *B. bassiana***	**In this study**


## Materials and Methods

### Microbial Strains and Culture Conditions

The wild-type strain *B. bassiana* ARSEF 2860 (designated WT herein) and its mutants were cultivated in Sabouraud dextrose agar [SDAY (4% glucose, 1% peptone, and 1.5% agar) plus 1% yeast extract] at 25°C in a light/dark cycle of 12:12 h for normal growth and conidiation and in Czapek-Dox agar (CZA; 3% sucrose, 0.3% NaNO_3_, 0.1% K_2_HPO_4_, 0.05% KCl, 0.05% MgSO_4_, and 0.001% FeSO_4_ plus 1.5% agar) or 1/4 SDAY (amended with 1/4 of each SDAY nutrient) for their responses to nutritional and chemical stresses at the same regime. *Escherichia coli* DH5α from Invitrogen (Shanghai, China) for plasmid propagation was grown in Luria-Bertani medium at 37°C.

### Bioinformatic Analysis of Three HSF Domain-Containing Proteins in *B. bassiana*

The Hsf1, Sfl1, and Skn7 sequences of *S. cerevisiae* were used as queries to search through in the *B. bassiana* genome database under the NCBI accession NZ_ADAH00000000 (Xiao et al., 2012). The located three orthologs were structurally compared through online blast analysis at https://blast.ncbi.nlm.nih.gov/Blast.cgi. Their sequences were aligned with the counterparts of other representative filamentous fungi for phynogenetic analysis with a neighbor-jointing method in MEGA7 software at http://www.megasoftware.net.

### Generation of *hsf1*, *sfl1*, and *skn7* Mutants

The same strategy for the deletion of *mas5* or *mdj1* and associated backbone plasmids (Wang et al., 2016, 2017) were used to delete *hsf1*, *sfl1*, *and skn7* (Gene ID: 19887609, 19887228, and 19884300, respectively) from the wild-type strain *B. bassiana* ARSEF2860 (designated WT herein). Briefly, the 5′ and 3′ coding/flanking fragments of each gene were amplified from the WT via PCR with paired primers (Supplementary Table S1) under the action of La*Taq* DNA polymerase (Promega, Madison, MI, United States) and inserted into p0380-bar. The resultant plasmid p0380-5′*x*-bar-3′*x* (*x* = *hsf1*, *sfl1*, *or skn7*) was transformed into the WT for targeted gene deletion by homogeneous recombination of the 5′ and 3′ fragments separated by *bar* marker through *Agrobacterium*-mediated transformation. Subsequently, the full-length coding sequence of each gene with flanking regions was cloned from the WT and ligated into p0380-sur-gateway to exchange for the gateway fragment. The new plasmid p0380-sur-*x* was ectopically integrated into the corresponding deletion mutant via the same transformation system. Putative deletion or complemented mutant colonies were screened in terms of the *bar* resistance to phosphinothricin (200 μg/ml) or the *sur* resistance to chlorimuron ethyl (10 μg/ml) on a selective medium and identified through PCR, quantitative real-time PCR (qRT-PCR) and Southern blotting analyses with paired primers and amplified probes (Supplementary Table S1). Positive deletion mutants were evaluated in parallel with the WT and complemented strains (control strains) in the following experiments of three replicates (cultures or samples from the cultures).

### Phenotypic Experiments

Aliquots of 1 μL 10^6^ conidia/mL suspension were spotted centrally onto the plates (9 cm diameter) of rich Sabouraud dextrose agar, minimal CZA, and modified CZA media with different carbon or nitrogen sources and availability. The modified media were prepared by deleting 3% sucrose (carbon starvation) or 0.3% NaNO_3_ (nitrogen starvation) from the standard CZA, replacing 3% sucrose with 3% of glucose, galactose, glycerol, fructose, trehalose, maltose, or acetate (NaAc) as sole carbon source, and replacing 0.3% NaNO_3_ with 0.3% of NaNO_2_ or NH_4_Cl as sole nitrogen source, respectively. After 8-day incubation at 25°C and 12:12 h, the mean diameter of each colony was estimated as a growth index of each strain on a given medium using two measurements taken perpendicular to each other across the center of the colony. Additionally, SDAY colonies initiated as above were incubated at 34°C and 12:12 h for 8 days, followed by measuring colony diameters as a growth index of each strain at the high temperature.

SDAY cultures for quantification of conidiation capacity were initiated by spreading aliquots of 100 μL 10^7^ conidia/mL suspension. From day 4 onwards during 7-day incubation at the same regime, three culture plugs (5 mm diameter) were taken daily from each plate using a cork borer. Conidia on each plug were released into 1 mL of 0.02% Tween 80 via thorough vibration. The concentration of conidia in the suspension was assessed with a hemocytometer and converted to the number of conidia per cm^2^ plate culture.

The viability of the conidia from the SDAY cultures was assessed as median germination time (GT_50_) for each strain to reach 50% germination at the same regime. Conidial thermotolerance and UV-B resistance were quantified as median lethal time (LT_50_; min) after exposure to a wet heat at 45°C for 0–120 min and median lethal dose (LD_50_; J/cm^2^) after exposure to UV-B irradiation (weighted 312 nm) at 0-0.8 J/cm^2^, as described previously (Wang J. et al., 2013). Conidial virulence was bioassayed on *Galleria mellonella* larvae by immersing cohorts of ∼35 larvae for 10 s in 30 ml aliquots of a 10^7^ conidia/mL suspension for normal cuticle infection and injecting 5 μL of a 10^5^ conidia/mL suspension was injected into the hemocoel of each larva in each cohort for cuticle-bypassing infection as described previously (Wang et al., 2016). All treated cohorts were maintained in Petri dishes for up to 10 days at 25°C and monitored daily for mortality records, followed by probit analyses of time-mortality trends for the estimates of median lethal time (LT_50_) for each strain against the model insect.

To assess hyphal sensitivity to cell wall perturbation, hyphal mass disks (5 mm diameter) were taken from the 3-days-old cellophane-overlaid SDAY cultures initiated by spreading 100 μL of a 10^7^ conidia/mL suspension per plate and attached centrally to the plates (9 cm diameter) of 1/4 SDAY (amended with 1/4 of each SDAY nutrient) alone (control) or supplemented with a gradient of Congo red (0.5–3 mg/mL), followed by 6-day incubation at 25°C. An effective concentration (EC_50_) for Congo red to suppress 50% of colony growth was estimated by modeling analysis of relative growth indices of each strain over the chemical gradient. The relative growth index was computed as a colony diameter ratio of each chemical concentration over the control (Wang J. et al., 2013). Conidial sensitivity of each strain to a sensitive concentration of Congo red (1 mg/mL) was assessed as the ratio of its germination percentage at the concentration over that in the control after 24-h incubation at 25°C.

Cell wall fragility of hyphae was assessed using a method of cell wall degradation (Zeng et al., 2012). Aliquots of ∼100 mg hyphal cells (fresh weight) were collected from the 2-days-old liquid cultures in SDB (agar-free SDAY) containing 10^6^ conidia/mL, washed twice with PBS (pH 7.0) and resuspended in 2 mL aliquots of 0.8 M sucrose containing 10 mg/mL of snailase and lysing enzymes (Sigma). After 6-h incubation at 37°C, the cell suspensions were kept in ice for termination of cell wall lysing. The concentration of protoplasts released from the hyphal cells of each suspension was quantified as an index of the cell wall fragility using a hemocycometer.

Carbohydrate epitopes on the surfaces of conidia were probed with the Alexa fluor 488-labeled lectins concanavalin A [ConA specific to α-glucose and α-N-acetylglucosamine (α-GlcNAc)], *Galanthus nivalis* (GNL lectin specific to mannose residues), and wheat germ agglutinin (WGA specific to β-GlcNAc and sialic acids) from Molecular Probes-Invitrogen and Vector Laboratories, respectively. Briefly, conidia were fixed in 3% formaldehyde for overnight at 4°C, washed three times in PBS, and resuspended in the buffer, followed by centrifugation. The pretreated conidia were then suspended in each lectin-binding buffer for 1 h labeling in darkness following the user’s guide. Unbound lectin was removed by washing repeatedly with the binding buffer. Fluorescent intensity in every 2 × 10^4^ labeled conidia was quantified on the flow cytometer FC 500 MCL (Beckman Coulter, California, United States) using an argon laser at the excitation/emission wavelengths of 488/530 nm. Each lectin assay included three conidial samples as replicates.

Hyphal sensitivities to osmotic and oxidative agents were assayed by incubating the hyphal mass disks on the plates of 1/4 SDAY alone (control) or supplemented with the gradients of NaCl (0.4–2 M), menadione (2–8 mM), and H_2_O_2_ (20–80 mM) and quantified as an EC_50_ for each chemical to suppress 50% colony growth, as described for Congo red.

### Transcriptional Profiling of Phenotype-Related Genes

For each strain, 100 μL aliquots of a 10^7^ conidia/ml suspension were spread onto cellophane-overlaid plates of SDAY alone or 1/4 SDAY supplemented with NaCl (0.8 M), followed by 3-day incubation at 25°C. For a heat shock treatment, the 3-days-old hyphal cultures were exposed to 40°C for 1 h. Total RNA was extracted from each of the cultures under the action of an RNAiso^TM^ Plus Reagent (TaKaRa, Dalian, China) and reversely transcribed into cDNA with PrimeScript^®^ RT reagent kit (TaKaRa), respectively. The experiments of quantitative real-time PCR (qRT-PCR) with paired primers (Supplementary Table S2) were performed under the action of SYBR^®^ Premix Ex Taq^TM^ (TaKaRa) to assess the transcripts of: (1) three HSF domain-containing genes, several conidiation-related genes and 28 HSP genes in three cDNA samples (10-fold dilution, the same below) derived from the normal SDAY cultures; (2) three HSF domain-containing genes and 28 HSP-coding genes in the cDNA samples derived from the heat-shocked SDAY cultures; and (3) five Na^+^-ATPase genes in the cDNA samples derived from the 1/4 SDAY cultures co-cultivated with NaCl. The transcript of the fungal γ-actin gene in each treatment was used as internal standard. Relative transcript level of each gene was calculated as the ratio of its transcript in the cDNA of each mutant over that in the cDNA of the WT strain using the 2^-ΔΔC_t_^ method (Livak and Schmittgen, 2001).

### Quantification of Antioxidant Enzyme Activities

Aliquots of 0.5 g hyphal mass from the 3-days-old SDAY cultures of each strain grown at 25°C were ground in liquid nitrogen, suspended in 50 mM phosphate buffer (pH 7.4), and centrifuged for 20 min by 16 000 ×*g* at 4°C. The protein concentration (mg/mL) in the supernatant was determined using a bicinchoninic acid (BCA) Protein Assay Kit (KeyGen, Nanjing, China). Total activity (U/mg) of superoxide dismutases (SODs) in the extract was assayed based on the inhibition of spontaneous autoxidation of pyrogallol. One unit of SOD activity was defined as the SOD amount required to inhibit 50% pyrogallol autoxidation rate. To assay total activity of catalases (CAT), the homogenized hyphal samples were suspended in 20 mM sodium phosphate buffer (pH 7.3), centrifuged as above, and filtered through a 0.22 mm Millex HPF PVDF membrane (GE Healthcare, Shanghai, China), followed by assessing the protein concentration with the BCA kit. Total catalase activity (U/mg) in the supernatant was assayed by reading the OD_240_ value indicative of H_2_O_2_ decomposition. One unit of CAT activity was defined as 1 μM H_2_O_2_ consumed per min.

### Statistic Analysis

All data from the phenotypic experiments of three replicates were subjected to one-factor (strain) analysis of variance, followed by Tukey’s honestly significant difference (HSD) test for the difference of each phenotype between each deletion mutant and two control strains.

## Results

### Bioinformatic Features of Three HSF Domain-Containing Orthologs in *B. bassiana*

Orthologs of Hsf1, Sfl1, and Skn7 (NCBI accession codes: EJP66657, EJP66923, and EJP69323 respectively) located in the genomic database of *B. bassiana* (Xiao et al., 2012) consist of 724, 539, and 585 amino acids with molecular masses of 78.8, 59.1, and 63.7 kDa, respectively, and share a conserved HSF domain at N-termini (Supplementary Figure S1a). Additionally, Hsf1 has a central Tmemb_cc2 domain typical for transmembrane and coiled-coil-2 proteins (Zhang et al., 2014) and a C-terminal SOG2 domain typical for the proteins of RAM signaling pathway involved in cell separation and cytokinesis (Nelson et al., 2003). Skn7 possesses an RR (response regulator) domain. Sfl1 has a unique mitogen-activated protein kinase (MAPK) docking site (KRGDIAGR at residues 119–126). In phylogeny, the sequences of Hsf1, Sfl1, and Skn7 in *B. bassiana* share an identity of 26–49% with one another and are much more identical to the counterparts of *Cordyceps* than those of other filamentous fungi and yeasts (Supplementary Figure S1b).

### Interrelationships of *Hsf1*, *Sfl1*, and *Skn7* and Their Contributions to Heat Tolerance

The expected recombinant events for the deletion and complementation of *hsf1*, *sfl1*, or *skn7* were confirmed by PCR and Southern blotting analyses (Supplementary Figure S2). The transcript of each deleted gene was undetectable in either normal or heat-shocked cultures of each deletion mutant in qRT-PCR, providing further evidence for the success of each deletion.

**FIGURE 1:**
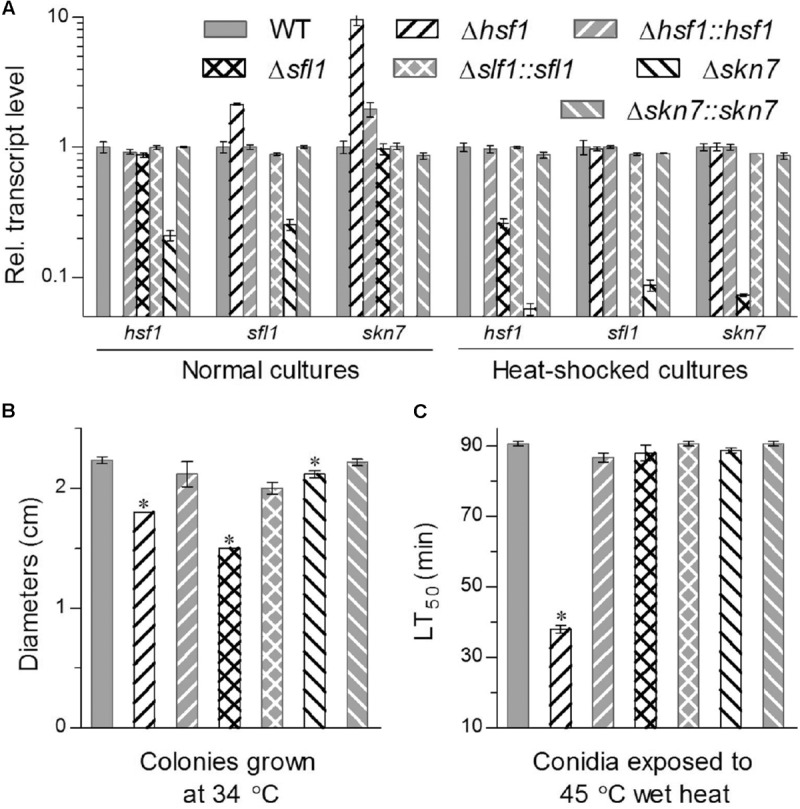
Deletion of *hsf1*, *sfl1* or *skn7* altered transcriptional profiles of two other genes and response to high temperature in *B. bassiana*. **(A)** Relative transcript levels of HSF domain-containing genes in the 3-day-old SDAY cultures of each mutant exposed or not exposed for 1 h to a heat shock at 40°C with respect to the WT standard. **(B)** Diameters of fungal colonies after 8 days of cultivation in SDAY at 34°C. Each colony was initiated by spotting 1 μL of conidial suspension. **(C)** LT_50_ (min) for conidial tolerance to a wet-heat stress at 45°C. Asterisked bars in each bar group differ significantly from those unmarked (Tukey’s HSD, *P* < 0.05). Error bars: SD from three replicates.

Intriguingly, deletion of one HSF domain-containing gene altered drastically transcript levels of two others in the 3-days-old SDAY cultures with respect to the WT standard under normal and heat-shocked conditions, as illustrated in **Figure 1A**. Deletion of *hsf1* resulted in an upregulation of *sfl1* and *skn7* by 1.1- and 8.5-fold under normal conditions, respectively, despite no impact on expression of either gene in response to a 1-h heat shock at 40°C. Transcript levels of *hsf1* and *skn7* in the Δ*sfl1* cultures were reduced respectively by 74 and 93% in response to the heat shock but not affected under normal culture conditions. In Δ*skn7*, expression of *hsf1* and *sfl1* was depressed respectively by 79 and 75% under normal conditions and 94 and 92% under the heat shock. These data indicated that the three HSF genes were conditionally interrelated at transcriptional level in *B. bassiana*. Particularly, the heat shock stimulated expression of *skn7* and *sfl1* in the absence of *hsf1* and depressed expression of *hsf1* and *skn7* in the absence of *sfl1*. Either *hsf1* or *sfl1* was highly sensitive to the absence of *skn7* irrespective of being exposed to the heat shock or not.

The three deletion mutants were differentially responsive to thermal stress. They grew significantly slower than paired control strains during 8-day incubation at the high temperature of 34°C, resulting in a decrease of mean colony size by 33% in Δ*sfl1*, 19% in Δ*hsf1*, and 5% in Δ*skn7* (**Figure 1B**). LT_50_ for conidial tolerance to a wet-heat stress at 45°C (**Figure 1C**) was shortened by 58% in Δ*hsf1* but did not differ significantly between two other mutants and their control strains (Tukey’s HSD, *P* > 0.05).

### Roles of *Hsf1*, *Sfl1*, and *Skn7* in Activating HSP Genes of Different Families

Transcript levels of all 28 HSP genes in *B. bassiana* were quantified in the 3-days-old SDAY cultures of each mutant versus the WT strain triggered with or without 1 h of heat shock at 40°C. As shown in Supplementary Table S3, significant changes were marked with red for the HSP genes upregulated (↑) by ≥ twofold or with green for those downregulated (↓) by ≥ 50% in each deletion mutant. The Δ*hsf1* mutant showed transcript levels of 13 HSP genes increased by 3- to 35-fold and of seven genes decreased by 50–94% under normal conditions while most of the upregulated genes were expressed at approximate WT levels or depressed significantly in response to the heat shock. Those showing remarked transcript changes in Δ*hsf1* under normal and heat-shocked conditions were *hsp20* (25% ↑ vs. 90% ↓), *hsp30a* (50% ↑ vs. 60% ↓), *hsp40b* (90% ↓ vs. 20% ↑), *hsp40c* (5-fold ↑ vs. 70% ↓), *hsp70b* (89% ↓ vs. 1.6-fold ↑), *hsp70g* (94% ↓ vs. 1.3-fold ↑), *hsp70h* (53% ↓ vs. 22% ↑), *hsp70k* (55% ↓ vs. 48% ↑), and *hsp104* (1.7-fold ↑ vs. 58% ↓). The normal and heat-shocked Δ*sfl1* cultures contained 10 and 23 HSP genes depressed by 50–88% and 50–98%, respectively. Striking transcript changes in the two types of Δ*sfl1* cultures were present in *hsp20* (37% ↑ vs. 89% ↓), *hsp40b* (32% ↑ vs. 89% ↓), *hsp40d*–*f* (WT level vs. 76–86% ↓), *hsp70a/c/d/j/k/m*–*o* (0.3- to 1.3-fold ↑ vs. 50–98% ↓), and *hsp104* (37% ↓ vs. 2.6-fold ↑). In Δ*skn7*, transcript levels of 18 HSP genes were reduced by 50–89% without the heat shock while 15 depressed (50–95%) HSP genes concurred with two significantly upregulated genes under the heat shock. The differentially expressed HSP genes in Δ*skn7* included *hsp30b* (75% ↓ vs. WT level), *hsp40a* (38% ↓ vs. 2.3-fold ↑), *hsp70b* (1.6-fold ↑ vs. 94% ↓), *hsp70g/h/k* (1.3- to 1.8-fold ↑ vs. 50–57% ↓), *hsp90* (50% ↓ vs. WT level), and *hsp78* (80% ↓ vs. 2.7-fold ↑). All of these transcript changes were restored to the WT levels by targeted gene complementation irrespective of being exposed or not exposed to the heat shock. These data implicated that, in *B. bassiana*, *Hsf1* was distinct from *Sfl1* and *Skn7* in activating most HSP genes under normal and heat-shocked conditions and that *Sfl1* and *Skn7* played overlapping roles in activating many HSP genes under heat shock although each activated preferentially a few HSP genes not targeted by two other HSFs.

### Roles of *Hsf1*, *Sfl1*, and *Skn7* in Vegetative Growth and Asexual Development

**FIGURE 2:**
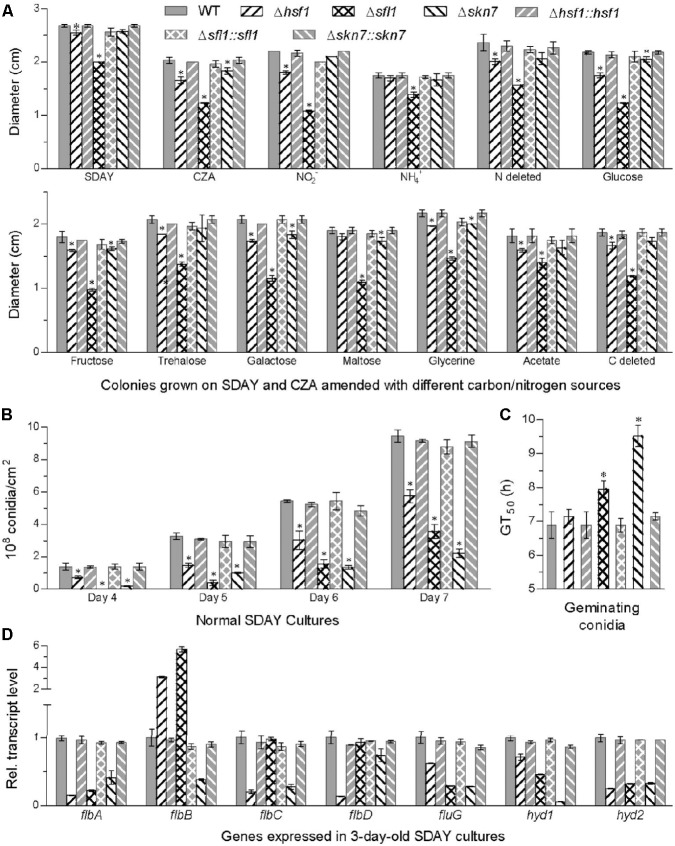
*Hsf1*, *Sfl1*, and *Skn7* play differential roles for in the asexual cycle of *B. bassiana*. **(A)** Diameters of fungal colonies after 8 days of cultivation at 25°C on rich SDAY, minimal CZA, and modified CZA media with different carbon/nitrogen sources and rich SDAY. Each colony was initiated with the spotting method. **(B)** Conidial yields quantified over the days of normal cultivation on SDAY plates spread with 100 μL aliquots of conidial suspension for culture initiation. **(C)** Median germination time (GT_50_) as an index of conidial germination rate. **(D)** Relative transcript levels of conidiation-required genes in the 3-day SDAY cultures of each mutant with respect to the WT standard. Asterisked bars in each bar group differ significantly from those unmarked (Tukey’s HSD, *P* < 0.05). Error bars: SD from three replicates.

Compared to WT, the mutants Δ*hsf1*, Δ*sfl1*, and Δ*skn7* displayed differential defects in vegetative growth, conidiation capacity and conidial germination. Most severe growth defects on rich SDAY, minimal CZA, and 11 CZA media modified with different carbon/nitrogen sources and availability were observed in Δ*sfl1*, whose colony sizes diminished by 21–51% after 8-day incubation on the media at the optimum 25°C (**Figure 2A**). Significant, but less severe, growth defects with no more than 18% decrease in colony size occurred in Δ*hsf1* grown on most media excluding NH_4_^+^ as sole nitrogen source and maltose as sole carbon source. The sizes of Δ*skn7* colonies diminished by no more than 12% on the nitrogen sources of NO_2_^-^ and NH_4_^+^ and the carbon sources of glycerol, glucose, fructose, galactose and maltose but were not significantly affected on other tested media (Tukey’s HSD, *P* > 0.05).

Conidial yields quantified from the 7-days-old SDAY cultures were averagely reduced by 76% in Δ*skn7*, followed by 62% in Δ*sfl1* and 39% in Δ*hsf1*, as compared with the WT yield (**Figure 2B**). Meanwhile, all the deletion mutants showed much fewer conidiophores and conidia than the control strains in microscopic examination of samples taken from colonies on day 3 (Supplementary Figure S3). Additionally, Δ*skn7* and Δ*sfl1* required longer time to achieve 50% of conidial germination at 25°C than the WT strain, but this difference disappeared in Δ*hsf1*, although germination percentages within 16 h were not affected significantly in all the mutants (**Figure 2C** and Supplementary Figure S3).

Intriguingly, transcript levels of several conidiation-required genes decreased drastically in the deletion mutants, including those encoding five transcription factors (FlbA–D and FluG) essential for activation of fungal development pathway (Etxebeste et al., 2010; Park and Yu, 2012) and two hydrophobins (Hyd1/2) crucial for conidial structure and hydrophobicity in *B. bassiana* (Zhang et al., 2011). As shown in **Figure 2D**, most of these gene transcripts decreased by 75–87% in Δ*hsf1* (*flbA/C/D* and *hyd2*), 54–77% in Δ*sfl1* (*flbA*, *fluG* and *hyd1/2*), and 58–94% in Δ*skn7* (all except *flbD*). The depressed expression of these genes in the deletion mutants could be causative of their conidiation defects, suggesting important roles for *Hsf1*, *Sfl1*, and *Skn7* in the asexual development of *B. bassiana*.

### Roles of *Hsf1*, *Sfl1*, and *Skn7* in Cell Wall Integrity

**FIGURE 3:**
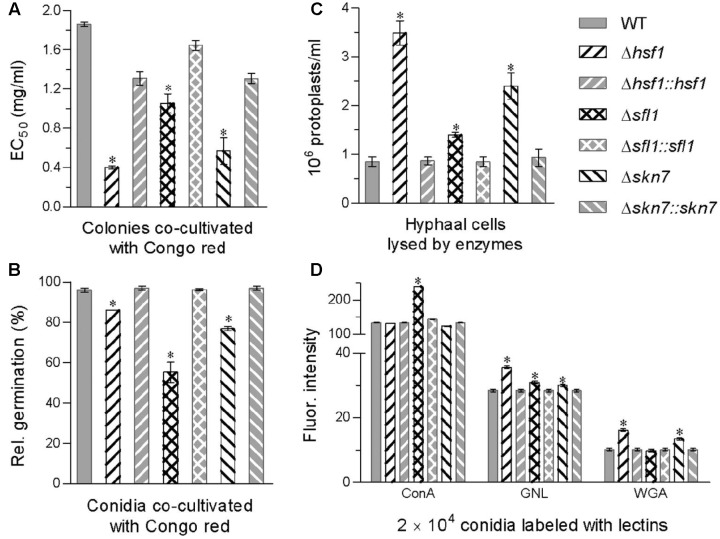
Contributions of *Hsf1*, *Sfl1*, and *Skn7* to cell wall integrity of *B. bassiana*. **(A)** EC_50_ for Congo red to suppress 50% colony growth of each strain after 6-day incubation at 25°C on 1/4 SDAY plates, to which small hyphal disks were attached for colony initiation. **(B)** Relative germination percentages of conidia after 24-h incubation at 25°C in a medium alone (control) or supplemented with Congo red (1 mg/mL). **(C)** Concentrations of protoplasts released from the hyphal cells after 6 h treatment with cell wall lysing enzymes in osmotic solution of 0.8 M sucrose. **(D)** Fluorescence intensity as an index of cell wall composition from flow cytometry of 2 × 10^4^ conidia labeled with the fluorescent lectins ConA, GNL, and WGA. Asterisked bars in each bar group differ significantly from those unmarked (Tukey’s HSD, *P* < 0.05). Error bar: SD from three replicates.

Cell wall integrity was differentially impaired by the deletions of *hsf1*, *sfl1*, and *skn7* in *B. bassiana*. First, an EC_50_ for the cell wall stressor Congo red to suppress 50% hyphal growth was lowered by 78% in Δ*hsf1*, 69% in Δ*skn7*, and 43% in Δ*sfl1* as compared with an estimate of 1.86 mg/mL from the WT (**Figure 3A**). Adding Congo red (1 mg/mL) to a medium suppressed conidial germination of the deletion mutants 10–42% more than that observed in the WT (**Figure 3B**). Second, more protoplasts were released from the hyphal cells of each deletion mutant than of the WT after 6 h cell lysing with enzymes (**Figure 3C**). The released protoplasts increased by 3.1-fold in Δ*hsf1*, followed by 1.8-fold in Δ*skn7* and 65% in Δ*sfl1*. These data indicated that hyphal cell walls became much more fragile in the absence of *hsf1* or *skn7* and of *sfl1*. Third, the deletion of each gene altered cell wall composition of conidia, as indicated by the data from the fluorescent lectin-binding assays (**Figure 3D**). The content of α-GlcNAc increased by 79% on the surfaces of ConA-labeled Δ*sfl1* conidia. The content of GNL-labeled mannose residues increased by 25, 9, and 6% on the surfaces of Δ*hsf1*, Δ*sfl1*, and Δ*skn7* conidia, respectively. The content of WGA-labeled β-GlcNAc increased by 59 and 32% on the surfaces of Δ*hsf1* and Δ*skn7* conidia, respectively. Finally, examination by transmission electron microscopy (TEM) revealed that an outermost layer of conidial wall in the deletion mutants was less distinctly outlined compared with the WT counterpart, making the mutant conidia readily deformed in the pretreatment for TEM (Supplementary Figure S4).

The increased cell wall sensitivity to Congo red, the increased cell wall fragility, the altered cell wall composition, and the less distinctly defined cell walls were largely or well restored by targeted gene complementation, indicating vital roles for *Hsf1*, *Sfl1*, and *Skn7* in sustaining the fungal cell wall integrity.

### Roles of *Hsf1*, *Sfl1*, and *Skn7* in Stress Tolerance and Virulence

**FIGURE 4:**
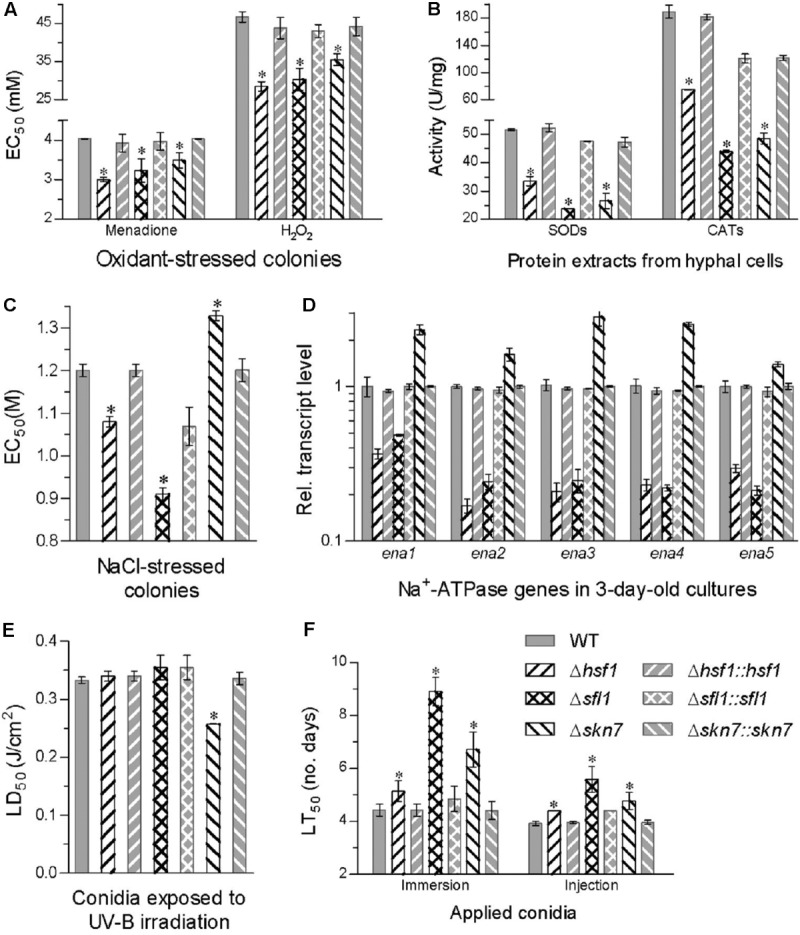
Deletion of each HSF domain-containing gene altered cellular sensitivity to oxidative, osmotic, and stresses, UV-B radiation and virulence. **(A,B)** EC_50_ estimates for menadione and H_2_O_2_ to suppress 50% colony growth after 6-day incubation at 25°C on 1/4 SDAY plates and total activities of SODs and CATs quantified in the protein extracts from 3-day-old SDAY cultures, respectively. **(C,D)** EC_50_ estimates for NaCl to suppress 50% colony growth on 1/4 SDAY and relative transcript levels of five Na^+^ ATPase genes in the 3-day-old 1/4 SDAY cultures of each mutant versus the WT strain co-cultivated with 0.8 M NaCl, respectively. **(E)** LD_50_ (J/cm^2^) estimates for conidial resistance to UV-B irradiation. **(F)** LT_50_ **(D)** for the virulence of each strain against *G. mellonella* larvae infected by topical application (immersion) or intrahemocoel injection of a standardized conidial suspension. Asterisked bars in each bar group differ significantly from those unmarked (Tukey’s HSD, *P* < 0.05). Error bar: SD from three replicates.

Compared to the WT strain, all deletion mutants became significantly more sensitive to two oxidants during colony growth (Tukey’s HSD, *P* < 0.05). The EC_50_ estimates for H_2_O_2_ and menadione to suppress 50% hyphal growth were lowered by 39 and 26% in Δ*hsf1*, 35 and 20% in Δ*sfl1*, and 24 and 13% in Δ*skn7*, respectively (**Figure 4A**). Their reduced antioxidant capabilities were consistent with reduced activities of SODs and CATs, which were quantified with the protein extracts from 3-days-old SDAY cultures. The SOD and CAT activity were reduced by 35 and 61% in Δ*hsf1*, 54 and 77% in Δ*sfl1*, and 48 and 74% in Δ*skn7*, respectively (**Figure 4B**). Interestingly, the EC_50_ values of menadione and H_2_O_2_ against all tested strains were linearly correlated with the total activities of their SODs (*r*^2^ = 0.71, *F*_1,5_ = 12.4, *P* = 0.017) and catalases (*r*^2^ = 0.72, *F*_1,5_ = 13.0, *P* = 0.016), respectively.

Hyphal sensitivity to NaCl increased by 24% in Δ*sfl1* and 10% in Δ*hsf1* but decreased by 11% in Δ*skn7* as compared with a mean EC_50_ of 1.2 M from the WT response to the salt (**Figure 4C**). The salt tolerance was closely linked to transcriptional changes of five Na^+^ ATPase genes (*ena1–5*) in the 3-days-old cultures co-cultivated with NaCl (0.8 M). Transcript levels of all five genes decreased by 64–83% in Δ*hsf1* and 52–79% in Δ*sfl1* but increased by 0.4- to 1.8-fold in Δ*skn7* with respect to the WT standard (**Figure 4D**). Additionally, only Δ*skn7* showed a remarked reduction (23%) in conidial tolerance to UV-B irradiation, as indicated by LD_50_ estimates from all tested strains (**Figure 4E**).

In standardized bioassays, a mean LT_50_ for the WT strain to kill 50% of *G. mellonella* larvae was 4.4 ± 0.2 days through topical application of 10^7^ conidia/mL suspension for normal cuticle infection and 3.9 ± 0.1 days through intrahemocoel injection for cuticle-bypassing infection (**Figure 4F**). Compared to the two means, the lethal action was significantly delayed in three deletion mutants irrespective of the infection through cuticular penetration or bypassing the insect cuticle (Tukey’s HSD, *P* < 0.05). The lethal action through normal infection was most delayed by 4.1 days in Δ*sfl1*, followed by a delay of 2.3 days in Δ*skn7* and 0.7 days in Δ*hsf1*. The delay of the lethal action through the cuticle-passing infection decreased to only 0.5–1.6 days in the three deletion mutants.

All of these changes were restored by targeted gene complementation, indicating that *Hsf1*, *Sfl1*, and *Skn7* play differential, but important, roles in sustaining antioxidant activity, salt tolerance, and virulence of *B. bassiana*.

## Discussion

Our study unveils that three HSF domain-containing genes are transcriptionally interrelated in *B. bassiana*. The drastic depressions of *hsf1* and *sfl1* in Δ*skn7* and of *hsf1* and *skn7* in Δ*sfl1* indicate that *skn7* or *sfl1* regulates expression of two other HSF genes positively under normal conditions and/or heat shock. In contrast, *hsf1* acts as a negative mediator of *sfl1* and *skn7* under normal conditions since its absence resulted in remarked upregulation of the two genes. The compensation of increased *skn7* and *sfl1* expression levels for the absence of *hsf1* provides an interpretation on the viability of Δ*hsf1* in *B. bassiana*, which might be inferred according to the observations in *S. cerevisiae* where deletion of *skn7* exacerbated the growth defect of the *hsf1* temperature-sensitive allele (*hsf1*^ts^) strain and high-copy expression of *skn7* rescued the growth defect of the *hsf1*^ts^ strain at 35°C (Raitt et al., 2000). This is very different from an indispensability of *hsf1* for *S. cerevisiae* and *N. crassa*, in which deletion of *hsf1* caused lethality (Wiederrecht et al., 1988; Thompson et al., 2008). Except for heat shock, Skn7 and Hsf1 might in some way cooperate in the response to free radical stress, which demonstrated again that there is clearly some overlap between the functions of *skn7* and *hsf1* in *S. cerevisiae* (Raitt et al., 2000).

Meanwhile, *Hsf1*, *Sfl1*, and *Skn7* played distinctive or overlapping roles not only in activating HSP genes in response to heat shock but also in regulating expression of some genes crucial for asexual development and intracellular Na^+^ homeostasis and perhaps many more associated with multi-phenotypic alterations but not examined in this study. Function loss of each resulted in increased sensitivity to 34°C during colony growth, largely in agreement with increased thermosensitivity in the absence of other fungal *hsf1* (Nicholls et al., 2011; Hamid et al., 2013), *sfl1* (Li et al., 2011), or *skn7* (Shang et al., 2015; Hussain et al., 2016). In *C. albicans*, *Hsf1* can activate *hsp104*, *hsp90*, and *hsp70* under normal and stressful conditions (Nicholls et al., 2009, 2011). *Sfl1* also mediates expression of *hsp30*, *hsp90*, and *hsp98* in *S. cerevisiae*, *C. albicans*, and *M. oryzae* under normal and/or stressful conditions (Galeote et al., 2007; Li et al., 2007, 2011; Zhang et al., 2008). The yeast *Skn7* and *Hsf1* can co-mediate expression of *hsp12*, *hsp26*, several *hsp70*, *hsp82*, and *hsp104* under oxidative stress (Lee et al., 1999; Raitt et al., 2000). In this study, each HSF domain-containing gene could specifically activate some HSP genes under normal or heat-shocked conditions. We found overlapping roles of *Sfl1* and *Skn7* in activating *hsp30b*, *hsp70f/e*, *hsp90*, and *hsp78* and of *Sfl1* and *Hsf1* in activating *hsp70i/l* but opposite roles of *Hsf1* and *Skn7* in activating *hsp20*, four *hsp40*, *hsp60*, and five *hsp70* genes in *B. bassiana* under normal conditions. In response to heat shock, *Sfl1* cooperated with *Skn7* for positive mediation of *hsp20*, *hsp40d*–*f* and nine *hsp70* genes or with *Hsf1* for positive mediation of *hsp30b*, *hsp40f*, *hsp70a*, and *hsp90* and opposite mediation of *hsp104*; *Skn7* cooperated with *Hsf1* for positive mediation of *hsp40c/f*. Regardless of more or less involvement in heat tolerance and regulating HSP genes alone or cooperatively, three HSF domain-containing genes were involved in physiological and cellular processes relevant to the fungal adaptation to environment and host, as discussed below.

First, three HSF domain-containing genes play important, but differential roles in sustaining the asexual cycle of *B. bassiana*. The growth defects of their deletion mutants on different media indicate that *Sfl1* is more important than *Hsf1* for the fungal pathogen to make use of carbon/nitrogen sources for vegetative growth. The importance of *Sfl1* for the fungal growth is different from what have been learned from some other fungi, in which Δ*sfl1* mutants could grow as well in rich medium as wild-type strains (Li et al., 2007, 2011; Thompson et al., 2008). The limited role of *skn7* in the fungal growth is similar to minor growth defect due to *skn7* deletion in *F. graminearum* (Jiang et al., 2015) but somewhat different from no obvious role of the same gene in other fungal growth (Viefhues et al., 2015; Hussain et al., 2016; Zhang et al., 2016). The role of *Hsf1* is dispensable for *B. bassiana*, unlike its indispensability for *S. cerevisiae* and *N. crassa* (Wiederrecht et al., 1988; Thompson et al., 2008). Aside from differential roles in nutritional exploitation and vegetative growth, three HSF domain-containing genes are all required for full conidiation in *B. bassiana* despite different contributions. Particularly, *Skn7* is most important for not only conidiation but also conidial viability, followed by *Sfl1* and *Hsf1*. Their roles in conidiation are consistent with those of *Hsf1* in *Coniothyrium minitans* (Hamid et al., 2013), *Sfl1* in *M. oryzae* (Li et al., 2011), and *N. crassa* (Thompson et al., 2008) and *Skn7* in other filamentous fungi (Jiang et al., 2015; Yang et al., 2015; Hussain et al., 2016; Zhang et al., 2016). Our transcriptional analyses indicate prominent links of three HSF domain-containing genes to most transcription factors, which are required for the activation of central developmental pathway to govern asexual development (Etxebeste et al., 2010; Park and Yu, 2012), and hydrophobins that ensure conidial surface structure and hydrophobicity (Zhang et al., 2011). We speculate that the regulatory roles of three HSF domain-containing genes in the fungal asexual cycle are likely associated with expression levels of those phenotype-related genes.

Moreover, various cellular events rely upon cell wall integrity in fungi, including glycosylation of proteins, biosynthesis of glycosyl- phoshatidylinositol (GPI) anchors, quality control of secretory proteins, and delivery of cell wall components for assembly (Lesage and Bussey, 2006; Scrimale et al., 2009). In this study, *Hsf1*, *Sfl1*, and *Skn7* were vital for the cell wall composition and integrity of *B. bassiana* due to altered cell wall components (α-GlcNAc, β-GlcNAc, and mannose residues), increased cell wall fragility, and higher sensitivity to cell wall perturbation in the absence of each. Our results are well in accordance with increased sensitivities of Δ*skn7* mutants to cell wall stressors, such as Congo red and/or Calcofluor white, in other filamentous fungi (Shang et al., 2015; Viefhues et al., 2015; Zhang et al., 2016) but are different from null response of Δ*skn7* mutants to cell wall stress in two yeasts (Singh et al., 2004; Fassler and West, 2011).

Furthermore, three HSF domain-containing genes also function in sustaining antioxidant activity of *B. bassiana*. This is indicated by reduced activities of intracellular SODs and CATs and increased cellular sensitivities to two typical oxidants in Δ*hsf1*, Δ*sfl1*, and Δ*skn7*. Total activities of SODs and CATs are crucial for *B. bassiana* responses to menadione and H_2_O_2_, respectively (Xie et al., 2012; Wang Z.L. et al., 2013; Li et al., 2015) and were linearly correlated to antioxidant responses of all tested strains in this study. This implicates that maintenance of antioxidant activity by the three HSF domain-containing genes relies upon their involvements in mediating expression of antioxidant enzymes. Previously, increased sensitivity to oxidative stress was also caused by deletion of *skn7* in yeast (Lee et al., 1999) or filamentous fungi (Lamarre et al., 2007; Viefhues et al., 2015; Yang et al., 2015; Hussain et al., 2016; Zhang et al., 2016) but not in *M. robertsii*, another fungal insect pathogen (Shang et al., 2015). In addition, transcriptional changes of five Na^+^ ATPase genes coincide well with reduced or increased tolerance of each deletion to NaCl in this study. Particularly, the increased tolerance of Δ*skn7* to NaCl is very different from null response or increased sensitivity of Δ*skn7* to osmotic salts in other fungi (Chen et al., 2012; Shang et al., 2015; Yang et al., 2015). Conidial thermotolerance and UV-B resistance crucial for *B. bassiana* survival in the field were also lowered in Δ*hsf1* and Δ*skn7*, respectively. Altogether, *Hsf1*, *Sfl1*, and *Skn7* play differential roles in the fungal adaptation to oxidative, ion osmotic, thermal, and UV-B irradiative stresses.

Finally, *Sfl1* contributes much more to the virulence of *B. bassiana* than *Skn7* and *Hsf1* based on delayed lethal action in the absence of each. The attenuated virulence of their deletion mutants is similar to what was caused by the deletion of *hsf1* (Nicholls et al., 2011; Hamid et al., 2013), s*fl1* (Li et al., 2011), or *skn7* (Singh et al., 2004; Wormley et al., 2005; Chen et al., 2012; Shang et al., 2015) in other fungal pathogens. However, these observations are different from little change in the Δ*skn7* virulence of *Aspergillus fumigatus* (Lamarre et al., 2007), *B. cinerea* (Yang et al., 2015), *F. graminearum* (Jiang et al., 2015), and *M. oryzae* (Motoyama et al., 2008). For the deletion mutants in this study, the degrees of attenuated virulence to the tested model insect via normal cuticle infection are largely attributable to partial losses of their ability to grow on scant insect integument for hyphal penetration through the host cuticle, as shown with their growth defects on minimal media, and reduced antioxidant activity. The latter phenotype is important for fungal ability to resist an oxidative stress generated from the host immunity defense and linearly correlated with virulence in *B. bassiana* (Xie et al., 2012, 2013).

## Conclusion

There exists three HSF domain-containing genes transcriptionally interrelated in *B. bassiana*, which are respectively homologous to *hsf1*, *sfl1*, and *skn7* in yeast and other fungi. Here, our results indicate that three HSF-containing genes play vital but differential roles in sustaining the asexual cycle, virulence, multiple stress tolerance, and activating different families of HSP genes in *B. bassiana*, which reveals possible means to improving field persistence and efficacy of a fungal formulation by manipulating the HSF domain-containing genes of a candidate strain. Additionally, further investigations will be needed to have a deeper insight into the interrelationships of three HSF domain-containing genes which might be not limited in the transcriptional level.

## Ethics Statement

This study does not involved any experiment with human participants or animal performed.

## Author Contributions

GZ and JW designed and performed the experiments, analyzed the data, and prepared the manuscript. S-HY and XF contributed to the manuscript revision. YH performed the experiments. M-GF contributed to the manuscript revision and overall support of this study.

## Conflict of Interest Statement

The authors declare that the research was conducted in the absence of any commercial or financial relationships that could be construed as a potential conflict of interest.
