# Supplementary material for: Roles of Three HSF Domain-Containing Proteins in Mediating Heat-Shock Protein Genes and Sustaining Asexual Cycle, Stress Tolerance, and Virulence in Beauveria bassiana
Source: Front Microbiol. 2018 Jul 25;9:1677. doi: 10.3389/fmicb.2018.01677 (PMC6068467; doi:10.3389/fmicb.2018.01677)
Supplement: Supplementary file 1 [file Presentation_1.pdf]

Roles of Hsf1, Sfl1 and Skn7 in mediating heat-shock protein genes and sustaining asexual cycle, stress tolerance and virulence in *Beauveria basssiana*

Gang Zhou[1,2,3], Sheng-Hua Ying[3], Yue Hu[3], Xiang Fang[1]*, Ming-Guang Feng[3]*, Jie Wang[1,3]*

[1] College of Food Science, South China Agricultural University, Guangzhou, Guangdong, 510642, China (Corresponding at E-mail: wangjielangjing@126.com)

2 Guangdong Open Laboratory of Applied Microbiology, Guangdong Provincial Key Laboratory of Microbial Culture Collection and Application, State Key Laboratory of Applied Microbiology Southern China, Guangdong Institute of Microbiology, Guangzhou, Guangdong, 510070, People's Republic of China

3 Institute of Microbiology, College of Life Sciences, Zhejiang University, Hangzhou, Zhejiang, 310058, China

Table S1. Paired primers used for the manipulation of *hsf1*, *sfl1* and *skn7* in *B. bassiana*.

Primer	Paired sequences (5′–3′)*	Purpose
Hsf1up-F/R	AAAAAGAATTCGCTGCGAGAGTCAAGAGAG / AAAAAAAGCTTGTGTCGGGTTGTGGGGTTAT	Cloning *hsf1* 5′-end (1045 bp)
Hsf1dn-F/R	AAAAACTCGAGAGGAGGAGAGCGTGGAGAAG / AAAAAACTAGTGGGGTTATGAACACCTCCAG	Cloning *hsf1* 3′-end (1072 bp)
Sfl1up-F/R	AAAAAGAATTCGGTAGATTGTAGCACAGACGGG / AAAAAGAGCTCACGAGGGTGGAAGTGGAATAAC	Cloning *sfl1* 5′-end (1583 bp)
Sfl1dn-F/R	AAAAATCTAGAGCTCGGTCCCAAAGAAAACA / AAAAAAGATCTCGAATCTGGCTCTCAGGTGTAT	Cloning *sfl1* 3′-end (1432 bp)
Skn7up-F/R	AAAAAGAATTCGGTAGCGACTGTGTTGTTGGC / AAAAACCCGGGGTTGACGACGGGGTGAAAAG	Cloning *skn7* 5′-end (2113 bp)
Skn7dn-F/R	AAAAAAGATCTATGCCGTAAGTTGCGTCGTC / AAAAAACTAGTCGTCGTAGGCTGTCTGCTGT	Cloning *skn7* 3′-end (2298 bp)
Hsf1fl-F/R	*GGGGACCACTTTGTACAAGAAAGCTGGGT*GACCAGCGTCACGGCAAGAAT / *GGGGACAAGTTT GTACAAAAAAGCAGGCT*GGACTGAATGCGACAGGCGTT	Cloning full-length *hsf1* (5390 bp)
Sfl1fl-F/R	*GGGGACCACTTTGTACAAGAAAGCTGGGT*GGGGAGAGAACAGATTTGGAC / *GGGGACAAGTTT GTACAAAAAAGCAGGCT*TCGTTGGACCTACTCCTTTTC	Cloning full-length *sfl1* (4336 bp)
Skn7fl-F/R	*GGGGACCACTTTGTACAAGAAAGCTGGGT*CTTAGCGATGTGAAGCAGGTT / *GGGGACAAGTTT GTACAAAAAAGCAGGCT*CAGAGCACCAACAAACAACAT	Cloning full-length *skn7* (5811 bp)
pHsf1-F/R	CAAGCACCTCACAACAACAAAG / CTCACGCTTGGCTTTGGCT	PCR detecting *hsf1*
pSfl1-F/R	CACTACACTTCGTCTTTACCGCCAG / TCGGGAGGCAGTTGAACAGGT	PCR detecting *sfl1*
pSkn7-F/R	TCGAACTCTTCTCTTCCGCG / TGGTCTCTTCCCACAGCATT	PCR detecting *skn7*
sbHsf1-F/R	TGTGACTCAAGGTGAAAAAAG / GAATATATTGTGGCAGTGGGT	Southern probe for *hsf1*
sbSfl1-F/R	GTAGGTAGGTATCACTTGCC / GCACTGGAGAGAGAAAAAAT	Southern probe for *sfl1*
sbSkn7-F/R	TTCTCCTCCTCATTCCGTCTT / GACACCGAAGGAGGTTGACAG	Southern probe for *skn7*
qHsf1-F/R	GTCCGATGAACCAGTCTT/AAAGGAGTCCAGGGATCT	qRT-PCR detecting *hsf1*
qSfl1-F/R	ATCCAGCATCTAATATCA/TAAAGTATTGGGAGAGAA	qRT-PCR detecting *sfl1*
qSkn7-F/R	GAACAATGCCAGTGAATT / CGACAACAACAAAGGTAT	qRT-PCR detecting *skn7*
qActin-F/R	TCTCTACGGCAACATTGTC / TGCTGGAAGGTGGATAGG	qRT-PCR detecting γ-actin gene

*Underlined regions denote the sites of restriction enzyme for the deletion of each target gene (*Eco*RI/*Hin*dIII and *Xho*I/*Spe*I for *hsf1*, *Eco*RI/*Sac*I and *Xba*I/*Bgl*II for *sfl1*, and *Eco*RI/*Xma*I and *Bgl*II/*Spe*I for *skn7*) via homogenous recombination of 5′ and 3′ fragments separated by *bar* marker or the gateway exchange fragments for complementation of each target gene.

Table S2. Paired primers used for assessing transcript levels of 46 genes in the mutants of *hsf1*, *sfl1* and *skn7* versus *B. bassiana* wild-type via qRT-PCR.

Gene ID	Gene	Annotation	Sequences (5'-3') of paired primers
**Involved in growth and asexual development**			
19887954	*fluG*	developmental protein	CCTCCCTAGTTTGGTCGCTTTCTC / CGCTGTCGGTAATCTGCTCCTC
19885980	*flbA*	developmental regulator	CCAATCCACTCGCCGCTCTC / CGGAGGAAAGAGAATCGGTAGAGG
19890000	*flbB*	bZIP transcription factor	GCACTGACACGCCGACAAGAGC / CCGCCGCCGAAGCCTGTTG
19886193	*flbC*	C_2H_2 conidiation transcription factor	TCCATCTCCAACTTGCTGGGTCTC / GGCGGCGTAGGCGGAAGG
19890271	*flbD*	MYB conidiophore development protein	CGGCAAGCGATGGGCAGAGATTG / ACGAGCAAGGTGACGGTAGAGGTG
19889611	*hyd1*	hydrophobin 1	TTCTCAGCGATCTTGATCTT / GCACTTGTTGTCGATTGG
19886027	*hyd2*	hydrophobin 2	CATGGTGGAAAGGATCTG / ATCTTGGTCGTCTTCTCG
**Involved in Na^+ efflux**			
19889202	*Ena1*	P-type Na^+/K^+ ATPase 1	TTCTCACCGACGATAACT / TATTCTCAGCCAGGACAT
19892062	*Ena2*	P-type Na^+/K^+ ATPase 2	TAATACCAGGCAAGAAGT / TTAGGACGACAATGAGAG
19886191	*Ena3*	P-type Na^+/K^+ ATPase 3	GAATCTCAGCAGACTCAT / AATCACGAATCTCTTCCT
19887969	*Ena4*	P-type Na^+/K^+ ATPase 4	CGATGATGACAGCAAGTT / CGATAGAGCACCACAGAT
19890673	*Ena5*	P-type Na^+/K^+ ATPase 5	CAAGGATACAAGCGGTAA / CGACGAAGTAATGAGGTT
**HSP-coding genes**			
19890898	*hsp20*	heat shock protein 20	TCAGGAGATGAGAAGAAC / GCTGTGATGATGTTTGTT
19891700	*hsp30a*	heat shock protein 30	TCTTCCCTTGGTCGTGAT/GTATTGGTCGCTGCTCTC
19885069	*hsp30b*	heat shock protein 30	ATGGCTTCCTTCTTTCCC/AGTATGTGTCAAACTCGTC
19886748	*hsp40a*	heat shock protein 40	GAGATTGACCTCTATGAG / CTCCTTGAATTTGACCTC
19891452	*hsp40b*	heat shock protein 40	AGAAGACTACTACAAGATCC / TTCCTTGGCAGTATCATC
19889942	*hsp40c*	heat shock protein 40	ATTTCACACGACGAACAAG / TGCGAGTCCATAGTAAGC
19888317	*hsp40d*	heat shock protein 40	CAGGTACTTTCCGACAAG / CGAAAATAGACGAGAAGAAC
19885590	*hsp40e*	heat shock protein 40	GACAAGAACAAGGACGAT / ACCTCTCAGCAGAAATTC
19883188	*hsp40f*	heat shock protein 40	AATCGGTGCTCTCAAGTT / TGTCGGAAAGAATTTCGTAAG
19888479	*hsp60a*	heat shock protein 60	TTTACATCTTCACTACGA/CCTTCTCTAATCAGTTCT
19892463	*hsp70a*	heat shock protein 70	GTGGTAAAGGCATGATTT / GAGTCAGAAGTTGGAAAG
19889799	*hsp70b*	heat shock protein 70	GGACCTATGGCTCAACAG / AATGCGACACCAGAAAAG
19891640	*hsp70c*	heat shock protein 70	TTGGCTCAGTTGAATGTG / GCAAGTCTGGAAAGCAATA
19892721	*hsp70d*	heat shock protein 70	TTCTTGTAGCCGTTGATT / GGTATTATCTCCTCCTTCAC
19884009	*hsp70e*	heat shock protein 70	AGCAGAAGCAATGTCAAT/TGGCTGATGAAGAAGTTG
19886620	*hsp70f*	heat shock protein 70	AGCCGTCTTCTTCTTCTC/GCCTTGATGTACTCTGTG
19883269	*hsp70g*	heat shock protein 70	CATGGTTCTCCTCAAGAT / CTGGTTGTCGTTGAAGTA
19888598	*hsp70h*	heat shock protein 70	TGCTGGTGGTATGATGAC / TCGGAGAAGGTAGAGAAGA
19886917	*hsp70i*	heat shock protein 70	ATTATGGCACTGTCATTG / TGATACGGTTTCCTTGAT
19885360	*hsp70j*	heat shock protein 70	ACATCAAGGTCACTAAGC / CTTCTCTTCGTCTTCGTC
19889859	*hsp70k*	heat shock protein 70	TCAACATTCACGGCATTC / CTTCTTGTCAGTGTCCAT
19884520	*hsp70l*	heat shock protein 70	TCTGTCCTTAACGATACC / TCACGAAGAGCAGTAATC
19883953	*hsp70m*	heat shock protein 70	ACTCTACTTCAATATGGC / TAGGTCTACTTCCTCAAG
19886284	*hsp70n*	heat shock protein 70	CATTGTTGTCTCTATTGACT / TCTCCTTGATCTCCACTA
19883808	*hsp70o*	heat shock protein 70	TACAATCTGACTGAAGAACATATC / ATCGTTGAGCCTGACATT
19889528	*hsp90*	heat shock protein 90	TTCTCTCCCTCATCATCAAC/CTTGTAGCGGACCTTGTC
19885295	*hsp104*	heat shock protein 104	CTTTCGCCACAATTTCAC/ACACCGATGTAGGTATCTT
19884587	*hsp78*	heat shock protein 78	ATACAGACCTGCCGCTAT/TATGTTCGTGTGACCATTG

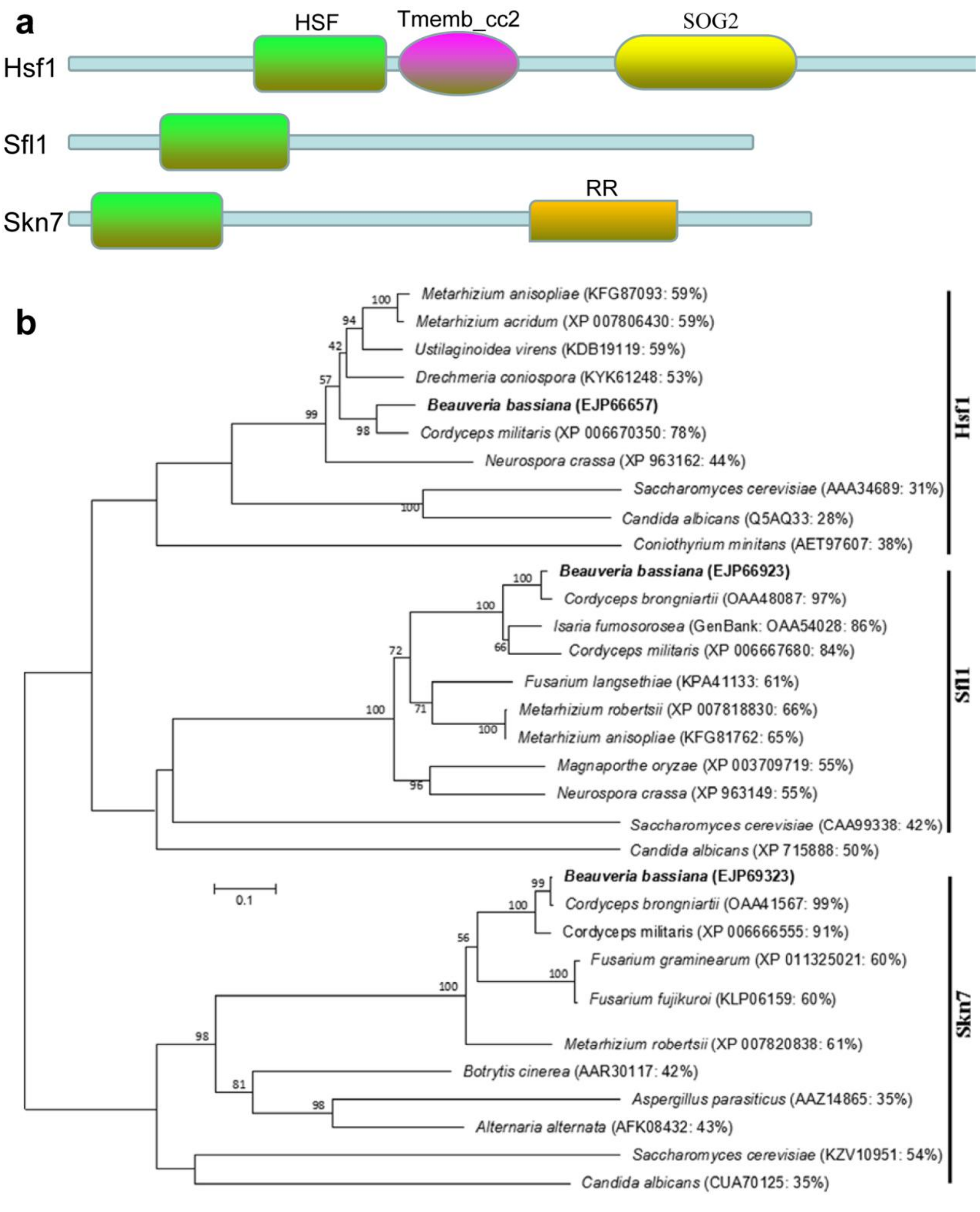

Fig. S1. Structural and phylogenetic features of three HSF-containing genes found in the genome database of *B. bassiana*. (**a**) Conserved domains predicted from three HSFs via sequence alignment and online blast analysis at http://blast.ncbi.nlm.nih.gov/blast.cgi. (**b**) Phylogenetic tree for three HSFs of *B. bassiana* and other fungi based on a neighbor-joining method in MEGA7 at http://www.megasoftware.net. The NCBI accession code of a given protein and its sequence identity to that of *B. bassiana* are given in the parentheses following each fungal name. Scale bar: branch length proportional to genetic distance.

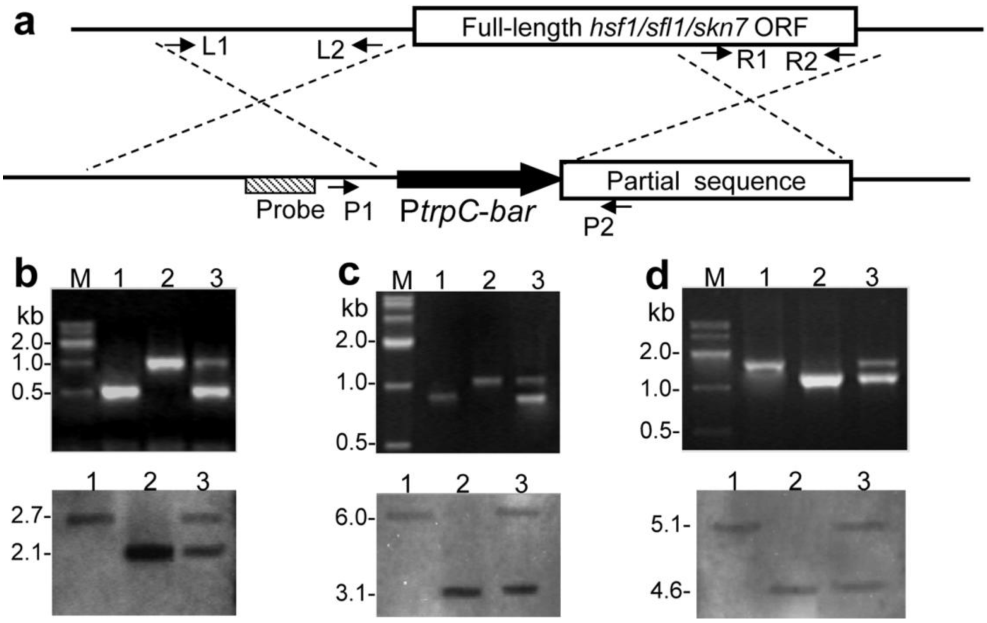

Fig. S2. Construction and identification of *B. bassiana hsf1*, *sfl1* and *skn7* mutants. (**a**) Schematic diagram for the deletion strategy of each target gene. (**b**–**d**) The *hsf1*, *sfl1* and *skn7* mutants identified via PCR (*upper panel*) and Southern blot hybridization (*lower panel*), respectively. *Lane 1*: wild-type strain. *Lane 2*: deletion mutant. *Lane 3*: complemented mutant. Genomic DNAs used for Southern blotting of *hsf1*, *sfl1* and *skn7* were digested with *Xho*I/*Xho*I, *Xba*I/*Xba*I and *Bst*XI/*Eco*RI, respectively.

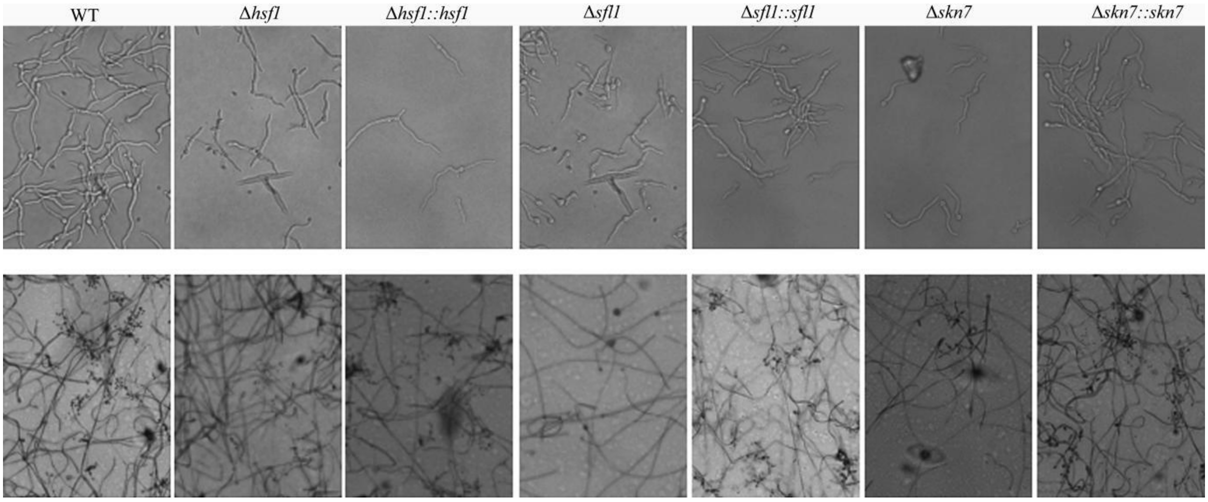

Fig. S3. Images of germ tubes and early hyphae after conidia were cultivated on GM plates for 16 h (up) and conidiophores and conidia after conidia were cultivated on SDAY plates for 3 days (down), visualized at 400× magnification.

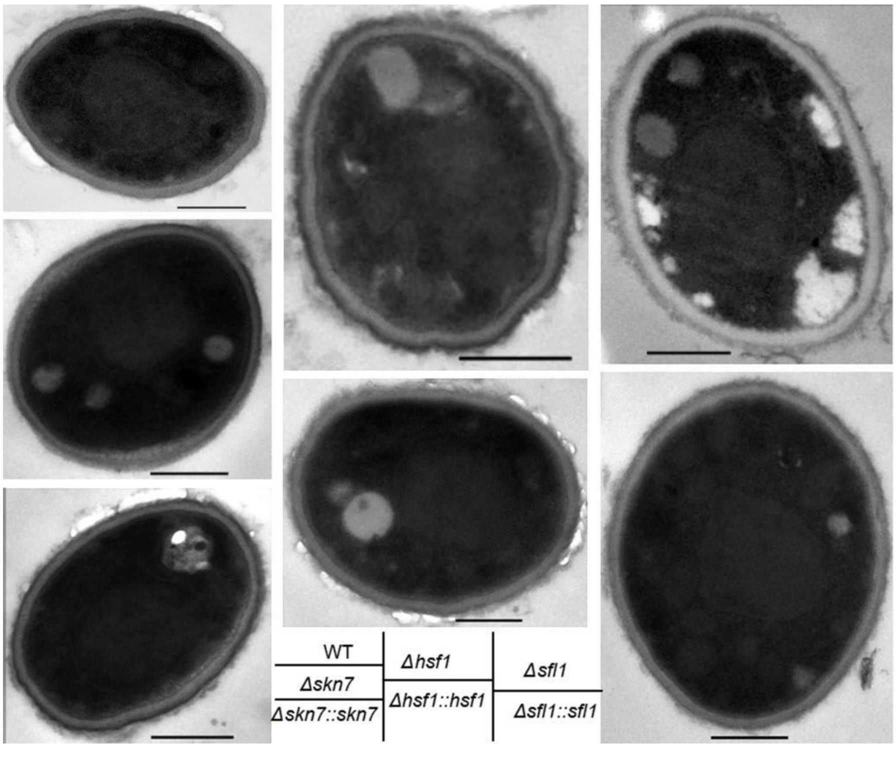

Fig. S4. Images for the ultrathin sections of conidia observed under transmission electron microscopy (TEM). Briefly, aerial conidia were washed three times in PBS (pH 7.0) and fixed overnight at 4 °C with 2% paraformaldehyde and 2.5% (v/v) glutaraldehyde in 0.1 M phosphate buffer (pH 7.2). After post-fixed in 1% OsO_4, conidia were dehydrated in a graded ethanol series, embedded in Spurr resin and stained with 2% uranyl acetate and Reynold's lead solution. The samples were examined under an H-7650 transmission electron microscope (Hitachi). Scale: 0.5 μm.

Table S3. Relative transcript levels of HSPs gene in the 3-d-old SDAY cultures of *hsf1*, *sfl1* and *skn7* mutants versus WT grown at 25°C or then shocked at 40°C for 1 h.

HSP genes	Relative transcript levels (mean ± SD) in normal cultures*							Relative transcript levels (mean ± SD) in heat-shocked cultures*						
	WT	Δ*hsf1*	Δ*hsf1::hsf1*	Δ*sfl1*	Δ*sfl1::sfl1*	Δ*skn7*	Δ*skn7::skn7*	WT	Δ*hsf1*	Δ*hsf1::hsf1*	Δ*sfl1*	Δ*sfl1::sfl1*	Δ*skn7*	Δ*skn7::skn7*
hsp20	1.00±0.09	13.5±0.97	1.71±0.35	1.38±0.11	1.02±0.08	0.24±0.01	0.97±0.02	1.00±0.07	2.14±0.03	1.00±0.02	0.11±0.02	0.95±0.04	0.47±0.03	1.00±0.02
hsp30a	1.00±0.09	1.25±0.14	0.97±0.13	0.83±0.02	1.00±0.02	0.58±0.01	1.00±0.01	1.00±0.03	0.10±0.01	0.95±0.03	0.78±0.02	0.97±0.04	0.54±0.02	0.95±0.03
hsp30b	1.00±0.11	1.50±0.10	1.02±0.06	0.23±0.02	0.96±0.02	0.25±0.01	0.96±0.03	1.03±0.31	0.43±0.04	1.00±0.03	0.09±0.01	0.93±0.06	0.94±0.02	1.00±0.02
hsp40a	1.00±0.06	0.17±0.01	1.00±0.01	1.03±0.06	1.02±0.06	0.62±0.03	0.93±0.04	1.04±0.39	0.54±0.03	1.00±0.02	0.79±0.04	1.00±0.03	3.34±0.33	1.35±0.39
hsp40b	1.00±0.10	0.10±0.00	0.95±0.04	1.33±0.02	1.01±0.02	0.45±0.02	0.96±0.04	1.01±0.14	1.21±0.04	1.00±0.03	0.17±0.04	0.95±0.03	1.14±0.07	1.00±0.05
hsp40c	1.00±0.06	6.30±0.31	1.63±0.35	1.09±0.02	0.99±0.02	0.24±0.01	0.99±0.01	1.00±0.02	0.30±0.01	1.00±0.01	0.61±0.05	1.00±0.03	0.14±0.01	0.93±0.10
hsp40d	1.01±0.13	7.45±0.67	1.71±0.47	1.03±0.07	1.02±0.06	0.19±0.01	0.95±0.04	1.00±0.12	1.07±0.02	1.00±0.01	0.14±0.01	0.99±0.01	0.07±0.00	0.87±0.01
hsp40e	1.01±0.14	8.99±0.90	1.74±0.18	1.57±0.17	1.11±0.02	0.20±0.02	0.95±0.04	1.00±0.04	0.84±0.03	1.00±0.02	0.21±0.00	1.00±0.00	0.12±0.00	0.84±0.03
hsp40f	1.00±0.12	5.53±0.12	1.67±0.50	0.95±0.02	1.00±0.02	0.28±0.00	0.97±0.03	1.00±0.08	0.49±0.04	0.97±0.03	0.24±0.01	1.00±0.01	0.18±0.00	1.00±0.00
hsp60	1.00±0.10	35.9±11.7	0.97±0.05	0.68±0.14	1.00±0.11	0.42±0.10	0.96±0.21	1.00±0.12	2.13±0.22	1.14±0.10	0.50±0.09	0.93±0.01	1.07±0.25	1.01±0.17
hsp70a	1.00±0.09	0.77±0.05	1.00±0.03	1.68±0.11	0.98±0.11	0.77±0.05	1.10±0.18	1.00±0.06	0.06±0.00	0.96±0.04	0.07±0.00	1.00±0.00	2.19±0.02	1.00±0.01
hsp70b	1.00±0.05	0.11±0.01	0.95±0.07	0.29±0.02	0.94±0.04	2.60±0.08	1.02±0.07	1.00±0.06	2.43±0.07	1.00±0.05	0.14±0.01	0.85±0.04	0.06±0.01	0.87±0.02
hsp70c	1.00±0.03	15.7±1.10	1.96±0.37	2.28±0.13	0.98±0.12	0.17±0.01	0.90±0.00	1.00±0.03	1.42±0.03	1.00±0.02	0.09±0.01	0.87±0.02	0.06±0.01	0.83±0.01
hsp70d	1.01±0.14	17.4±0.83	1.84±0.07	1.74±0.14	1.02±0.09	0.74±0.03	1.01±0.02	1.00±0.05	1.82±0.01	1.00±0.01	0.07±0.00	0.95±0.05	0.06±0.01	0.85±0.03
hsp70e	1.00±0.10	0.92±0.02	1.00±0.02	0.16±0.00	0.97±0.06	0.20±0.01	0.94±0.06	1.09±0.50	0.83±0.08	1.00±0.05	0.13±0.01	0.90±0.01	0.34±0.06	1.00±0.04
hsp70f	1.00±0.10	0.90±0.03	1.00±0.03	0.16±0.01	0.94±0.05	0.15±0.00	0.94±0.05	1.00±0.04	0.91±0.04	1.00±0.03	0.12±0.01	0.90±0.00	0.06±0.00	0.95±0.04
hsp70g	1.00±0.08	0.06±0.00	1.00±0.00	0.51±0.06	0.95±0.02	2.82±0.04	0.99±0.03	1.00±0.08	2.32±0.08	1.00±0.06	0.09±0.01	0.88±0.03	1.38±0.14	1.03±0.06
hsp70h	1.00±0.07	0.59±0.07	0.95±0.00	0.33±0.03	1.00±0.02	2.60±0.08	0.98±0.06	1.00±0.02	0.53±0.03	1.00±0.02	0.48±0.01	1.00±0.00	0.45±0.07	1.00±0.05
hsp70i	1.00±0.03	0.47±0.01	1.00±0.01	0.12±0.01	0.96±0.04	2.78±0.22	0.98±0.02	1.00±0.06	1.22±0.03	1.00±0.02	0.17±0.01	0.98±0.02	0.52±0.04	1.00±0.03
hsp70j	1.00±0.06	4.82±0.24	1.62±0.36	2.01±0.03	0.99±0.03	0.44±0.01	0.96±0.02	1.00±0.05	1.84±0.17	1.00±0.12	0.12±0.01	0.90±0.01	0.07±0.00	0.87±0.02
hsp70k	1.00±0.06	4.17±0.23	1.03±0.15	1.31±0.11	0.98±0.11	2.30±0.11	1.03±0.11	1.00±0.08	0.81±0.04	1.00±0.03	0.52±0.02	1.00±0.01	0.42±0.04	1.00±0.03
hsp70l	1.00±0.11	0.46±0.09	0.96±0.01	0.15±0.03	0.84±0.03	2.39±0.08	1.02±0.08	1.00±0.09	1.48±0.04	1.00±0.03	0.43±0.03	1.00±0.02	0.74±0.09	1.01±0.16
hsp70m	1.00±0.10	17.7±0.71	1.71±0.36	1.41±0.12	1.09±0.06	0.11±0.01	0.97±0.05	1.00±0.04	1.01±0.00	1.00±0.00	0.06±0.00	0.87±0.04	0.06±0.00	1.00±0.00
hsp70n	1.00±0.01	15.4±0.75	1.70±0.19	1.07±0.04	1.01±0.04	0.38±0.04	0.94±0.02	1.00±0.07	0.71±0.03	1.00±0.02	0.03±0.00	0.86±0.03	0.92±0.01	1.00±0.01
hsp70o	1.01±0.15	14.1±0.78	1.69±0.12	1.37±0.08	1.02±0.06	0.12±0.00	0.98±0.04	1.00±0.05	0.98±0.05	1.00±0.04	0.06±0.00	0.84±0.03	0.05±0.00	0.94±0.07
hsp90	1.00±0.02	0.75±0.02	1.01±0.02	0.33±0.01	0.99±0.02	0.54±0.01	1.00±0.00	1.00±0.07	0.23±0.02	1.00±0.01	0.48±0.04	0.97±0.03	1.06±0.04	1.00±0.02
hsp104	1.00±0.04	2.73±0.21	0.98±0.12	0.63±0.02	1.00±0.02	0.36±0.00	1.00±0.00	1.00±0.09	0.42±0.05	1.00±0.04	3.61±0.39	1.46±0.09	0.57±0.04	1.00±0.03
hsp78	1.00±0.00	0.93±0.00	1.00±0.00	0.15±0.00	0.90±0.00	0.20±0.01	0.80±0.01	1.00±0.08	0.66±0.01	1.00±0.01	0.63±0.03	0.97±0.03	3.71±0.86	1.44±0.13

* Red: HSP genes significantly upregulated by ≥ 2-fold. Green: HSP genes significantly downregulated by ≥ 50%.
